# Assessing the Utility of Deltoid Ligament Repair in Ankle Fracture: A Systematic Review

**DOI:** 10.7759/cureus.27040

**Published:** 2022-07-19

**Authors:** Kiya Shazadeh Safavi, Aryan Rezvani, Cory F Janney, Jie Chen, Waleed Cassis, Navid Darayan, Vinod K Panchbhavi, Daniel C Jupiter

**Affiliations:** 1 Department of Orthopedic Surgery and Rehabilitation, University of Texas Medical Branch at Galveston, Galveston, USA; 2 College of Medicine, Texas A&M Health Science Center, Bryan, USA; 3 Department of Orthopedic Surgery, Naval Medical Center San Diego, San Diego, USA; 4 College of Medicine, John Sealy School of Medicine, Univeristy of Texas Medical Branch at Galveston, Galveston, USA; 5 Department of Anesthesiology, Baylor College of Medicine, Houston, USA; 6 Department of Preventive Medicine and Population Health, University of Texas Medical Branch at Galveston, Galveston, USA

**Keywords:** ankle instability, syndesmosis, repair, ankle fractures, medial collateral ligament, deltoid, deltoid ligament

## Abstract

Ankle fractures are common injuries treated by orthopedists. Indications for operative repair of deltoid ligament (DL) injuries in ankle fracture patients are debated. The purpose of this review is to determine the indications for operative DL repair.

Ovid MEDLINE, CINAHL, and Scopus were searched up to December 2019. Web of Science was searched up to August 2018. Search terms included “Deltoid” and “Ligament” or “Ligaments.” Comparative studies assessing conservative vs operative DL repair were searched for. Articles meeting inclusion criteria were screened in two stages to determine eligibility.

Out of 1,542 articles, nine were included in our qualitative synthesis. These nine studies included 449 patients, of which 233 were treated with open reduction internal fixation (ORIF) with or without trans-syndesmotic (TS) screw fixation, and 205 of which were treated with ORIF with DL repair. The remaining 21 patients were managed nonoperatively, had no evidence of DL injury, or were lost to follow-up.

There is a lower rate of malreduction associated with DL repair compared to TS screw fixation. Moreover, DL repair may be useful in treating patients with Weber Type C fractures, concomitant DL-syndesmotic disruption, or residual valgus instability following ORIF in isolated lateral malleolar fractures.

## Introduction and background

Ankle fractures are among the most prevalent injuries treated by orthopaedic surgeons, with an incidence rate varying from 71 to 187 per 100,000 person-years [1]. Furthermore, up to 40% of acute fractures have an associated deltoid ligament (DL) injury [2].

The ideal approach for managing DL injuries in ankle-fracture patients has long been a subject of debate. Some argue for routine repair of DL injuries, while others assert that it has limited utility. Because the incidence of ankle fractures is expected to rise [3,4], detailed investigations into the role of DL repair in managing ankle fractures have become increasingly important.

**Figure 1:**
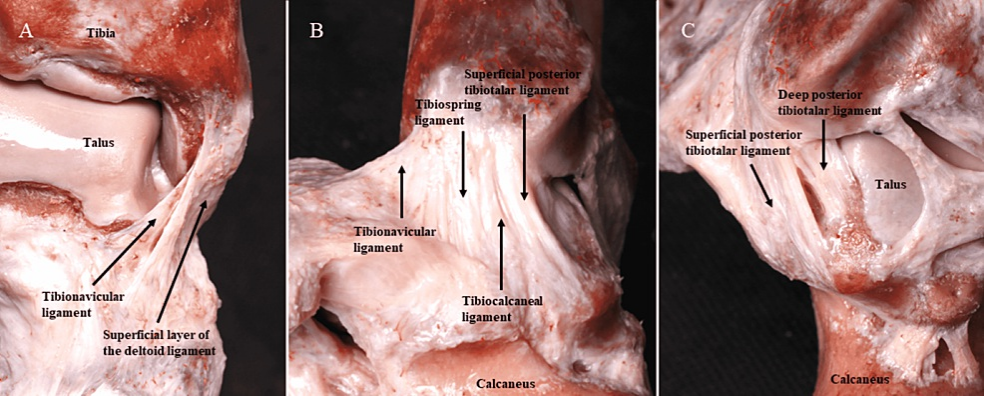
Anatomical dissection of the deltoid ligament showing (A) anterior, (B) lateral, and (C) posterior views of the deltoid ligament complex. These images are original and were created by the authors of this study.

Anatomically, the DL is a multifascicular complex, spanning the medial malleolus, talus, calcaneus, and navicular bone [5-7]. It is divided into superficial and deep layers [5-9]. The superficial layer is composed of the tibiospring, tibionavicular, superficial posterior tibiotalar, and tibiocalcaneal ligaments, and crosses the tibiotalar and subtalar joints. The deep layer includes the deep anterior tibiotalar and posterior tibiotalar ligaments and only crosses the ankle joint (Figures 1A-1C). Functionally, the superficial layer resists valgus stress, the deep layer acts against lateral talar displacement, and both layers prevent talar external rotation [6]. Additionally, recent cadaveric studies indicate that the DL is an indirect stabilizer of the syndesmosis [10-12].

Several diagnostic modalities are available for evaluating ankle fractures and DL integrity. Manual and gravity stress tests and magnetic resonance imaging (MRI) are reliable for evaluating supination-external rotation (SER)-type ankle fractures [13-16]. Other techniques include arthroscopy and ultrasonography [17]. Despite the known importance of the DL, consensus on optimal treatment options regarding DL repair in the setting of acute ankle fractures has yet to be determined. The purpose of this review is to examine the operative indications for DL repair in the setting of acute ankle fractures and to assess whether repairing an injured DL improves clinical outcomes in these cases.

## Review

Methods

General Statement

This study was performed according to guidelines set by Moher et al. in Preferred Reporting Items for Systematic Review and Meta-Analysis Protocols (PRISMA-P) 2015 statement [18].

Search Methods

An electronic literature search was performed using four databases (Web of Science, Ovid MEDLINE, CINAHL, and Scopus). Search terms “Deltoid” and “Ligament” or “Ligaments.” For databases with adjacency-searching capabilities, articles with the term “Deltoid” adjacent to within seven terms of “Ligament” were searched for. Each database was searched for articles published up to December 2019 except for Web of Science, which was searched for articles leading up to August 2018. Articles not published in English were excluded.

Articles meeting our search criteria were screened in two different stages. At each stage, two reviewers individually assessed articles to ensure interobserver reliability. When consensus was not achieved, a third reviewer determined whether to include an article. At the first screening stage, papers were reviewed and assessed using title and abstract alone. Papers dealing with the management of acute DL injury in the setting of ankle fractures proceeded to the second stage of screening, where they were read in full and evaluated. Studies were included if they contained comparative data on operative versus nonoperative DL treatment in patients with DL injury in the setting of closed ankle fractures. In these papers, we analyzed radiologic, functional, and clinical outcomes as well as post-operative complications and the need for reoperation. Abstract presentations, case reports, studies with one treatment group, and those not comparing quantitative or qualitative data regarding direct primary repair of the DL versus non-repair in patients with DL injuries were excluded. 

Data Extraction

Each included article was reviewed thoroughly by two reviewers separately to ensure accuracy and interobserver reliability of collected data. Relevant data regarding interventions, outcomes, conclusions, and limitations were manually extracted by the reviewer.

Statistical Analysis

No metanalyses or statistical analysis was performed in this study due to the broad and seldomly overlapping measured data points between papers used.

Results

**Figure 2:**
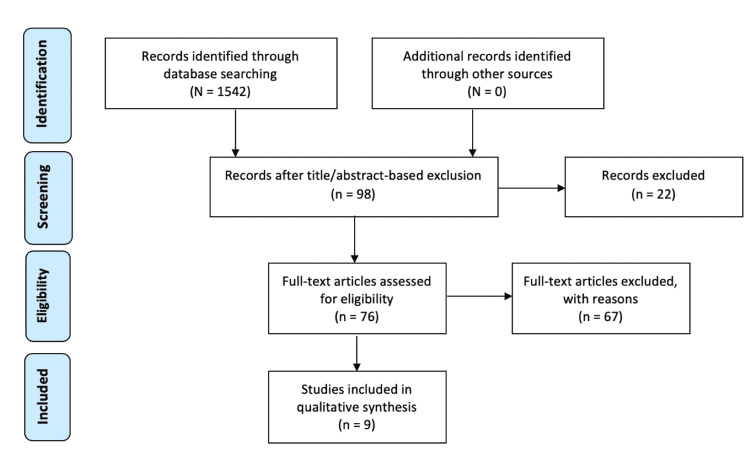
PRISMA flow diagram showing article selection process This figure was created using the PRISMA 2009 Flow Diagram. 1,542 papers were found. Screening based on article title and abstract, we excluded 1,444, leaving 98. Of these, 22 duplicates were excluded, leaving 76. Screening full manuscripts that discussed the DL another 67 were excluded as they were not specifically related to our topic, leaving nine to be used in our qualitative synthesis

We found 1,542 research papers. After screening based on article title and abstract, we excluded 1,444 articles that did not discuss the DL, leaving 98. Of these, 22 duplicates were excluded, leaving 76. After reading full manuscripts that discussed the DL, we excluded another 67 as they were not specifically related to our topic (non-operative vs operative fixation of the DL in the setting of acute ankle fractures), leaving nine for our qualitative synthesis (Figure 2).

Studies Supporting Routine DL Repair

Gu et al. evaluated 40 ankle-fracture patients with evidence of DL injury (medial clear space [MCS] > 5 mm and MRI confirmation). The patients were randomized into two groups of 20. One group underwent open reduction internal fixation (ORIF) alone (control group), and the other, ORIF and DL repair with suture anchors (treatment group). Observed indicators of efficacy included American Orthopaedic Foot and Ankle Scores (AOFAS), visual analog scale (VAS) scores, and postoperative MCS measurements. Mean follow-up times were 13.28 and 12.83 months in the control and treatment groups, respectively. Comparing the two groups, no significant difference was noted in AOFAS scores or MCS measurements, but VAS scores were significantly lower in the treatment group. Operation time and blood loss were increased in the treatment group (P = 0.026 and P = 0.032); however, hospital stays were significantly shortened (P = 0.041). No statistically significant differences in complication rates were found between the groups. The authors concluded that ORIF with DL repair can promote recovery of ankle function and alleviate pain with a remarkable curative effect and that DL repair should be popularized and routinely applied [19].

Woo et al. performed a retrospective comparative case series involving 78 patients with SER and pronation-external rotation (PER) lateral malleolar fractures and DL injury (as evidenced by lateral talar subluxation and/or MCS widening on radiography). Thirty-seven patients underwent ORIF, while 41 were treated with ORIF and DL repair. Patients from each group with evidence of syndesmotic instability were also treated with syndesmotic screw fixation. Intraoperative fluoroscopy, using stress gravity view, and external rotation tests assessed for residual instability after ORIF. Only patients with evidence of residual instability underwent DL repair. All patients followed a standardized rehabilitation protocol. The average follow-up time was 17 (range, 12-48) months. Average final follow-up MCS was smaller in the group that underwent ORIF and DL repair (P < 0.001). Radiographic evidence of medial instability at final follow-up was found in 11 patients (35.3%) treated with ORIF and 1 patient (2.4%) treated with ORIF and DL repair (P = 0.001). However, no significant clinical differences were described between the groups. In patients who had undergone concurrent syndesmotic fixation, average MCS was smaller in the DL-repair group (P < 0.02), and radiographic evidence of medial instability was present in six ORIF-only patients (P = 0.006). In the setting of syndesmotic fixation, clinical outcomes were superior in the DL-repair group. The authors concluded that medial instability may persist after fixation of ankle fracture and syndesmotic injury and that when ankle fracture and syndesmotic injury are accompanied by DL rupture, direct open repair of the DL (with ankle and syndesmotic fixation) may prove beneficial [20].

Zhao et al. retrospectively reviewed 74 closed Weber Type B and C fractures in patients with signs of DL injury (MCS ≥ 6 mm) [21]. Twenty patients underwent malleolar ORIF and DL repair via bone sutures enhanced with suture anchors. Fifty-four patients were treated with malleolar ORIF alone. All patients followed the same rehabilitation protocol. The authors did not distinguish the Danis-Weber classification types of patients who underwent syndesmotic fixation. MCS (preoperative, postoperative, and final follow-up), AOFAS, and VAS scores were assessed. Mean follow-up was 53.7 (range, 14-97) months. No malreductions (MCS ≥ 5 mm at any follow-up) or treatment failures (symptomatic malreduction) occurred in the DL-repair group. Malreduction occurred in 11 patients (20.4%) in the non-repair group, of whom seven had Weber Type C and 4 had Weber Type B fractures. Four patients (7.4%) in the non-repair group experienced treatment failure. Three had Weber Type C fractures and one had a Weber Type B fracture. The DL-repair group had significantly smaller postoperative and final follow-up MCS measurements (P = 0.03). No difference in AOFAS and VAS scores was noted between the two groups. Furthermore, the malreduction rate in Weber Type C patients who had not undergone DL repair was significantly higher than both unrepaired Type B patients and repaired Type C patients. They concluded surgical repair of the DL decreases the postoperative MCS and malreduction rate, especially for Weber Type C fractures [21].

Wu et al. evaluated 59 fractures with suspected DL injury to assess the utility of the intraoperative tap test of the distal tibiofibular syndesmosis in diagnosing DL rupture, and to compare the outcomes of trans-syndesmotic (TS) screw fixation to DL repair with suture anchor [22]. The tap test was positive in 53 cases, but surgical exploration revealed only 51 of these cases with combined DL injury and fracture. The sensitivity and specificity of the tap test were determined to be 100.0% and 75.0%, respectively. Of the 51 cases with DL injury and fracture, three were lost to follow-up and excluded from the study. The remaining 48 patients had Weber Type B or C fractures, evidence of DL injury (MCS > 4 mm and talar outward shift), and were randomized into two groups. Twenty-six patients were treated with ORIF and screw fixation and 22 with ORIF and DL repair. Mean follow-up time was 23.4 (range, 19-27) months for the syndesmosis repair group and 22.7 (range, 18-26) months for the DL-repair group. No statistically significant differences were found in AOFAS, VAS, and Short-Form (SF-36) Health Survey scores. Nine patients (34.6%) in the syndesmosis-repair group had syndesmotic malreduction, three of whom developed a syndesmotic screw fracture before the three-month postoperative mark. In the DL-repair group, two patients (9.09%) had a wide syndesmotic gap. The remaining 22 patients showed no notable changes in the anterior and posterior fibular incisura when comparing affected and unaffected ankles on postoperative computed tomography scans. The authors concluded that DL repair via suture anchor provided good functional and radiological outcomes comparable to syndesmotic screw fixation, and that syndesmosis screw fixation had a greater malreduction rate and increased risk of screw breakage [22].

Jones et al. performed a retrospective study on 27 patients with SER-IV bimalleolar equivalent fractures with MCS > 5 mm on stress testing [23]. They compared outcomes in patients treated with lateral malleolar ORIF and TS fixation to patients treated with lateral malleolar ORIF with DL repair. Fifteen patients were in the syndesmotic-repair group, and 12 were in the DL-repair group. Measured outcomes included AOFAS, VAS, Lower Extremity Function Scale, Foot and Ankle Disability Index, Foot and Ankle Outcome Score, Short Musculoskeletal Function Assessment, and function of the affected extremity. The mean follow-up time was 60.8 months for the syndesmosis-fixation group and 77.6 months for the DL-repair group. At final follow-up, 14 of 15 patients (93%) in the syndesmosis-fixation group, and 12 of 12 (100%) in the DL-repair group showed maintenance of anatomical mortise reduction and no signs of arthritis. No clinically significant differences could be found when comparing the two groups (using subjective questionnaires). All patients in the syndesmosis-fixation group required an additional operation for the removal of symptomatic syndesmosis implants. Moreover, there were two complications in that group that required repeat operative intervention (wound dehiscence requiring surgical debridement and a malreduced syndesmosis requiring further ORIF). No patients in the DL-repair group required reoperation due to conditions or symptoms related to the study. They concluded that, for SER-IV bimalleolar-equivalent ankle fractures, DL repair can restore congruity to the ankle joint and has subjective, functional, and radiographic outcomes comparable to syndesmotic fixation, while avoiding the risk of reoperation due to symptomatic implants [23].

Lee et al. performed a prospective analysis to assess the anterior DL’s contribution to stability against valgus force in patients with isolated lateral malleolar fractures [24]. They examined 35 SER Weber Type B lateral malleolar fracture patients with evidence of anterior deltoid (tibionavicular and tibospring) ligament and posterior deltoid (deep posterior tibiotalar) ligament damage. Patients were classified as being stable (Group S, n = 10) if they had a fracture displacement of < 2 mm, no evidence of shortening or rotation, talar tilt of one to two degrees, and MCS < 5 mm on the valgus stress test. Stable patients were treated conservatively with closed reduction and short leg casts. Patients not meeting the aforementioned criteria underwent lateral malleolar ORIF. Intraoperative fluoroscopy with valgus stress test was performed after fixation. Patients with no radiographic evidence of valgus instability were classified as being low-grade unstable (Group L, n = 15) and required no further operative treatment. Those with evidence of residual valgus instability were classified as high-grade unstable (Group H, n = 10) and underwent additional anterior deltoid repair. Measured outcomes included MCS measurements and MRI to evaluate for evidence of residual DL injury. At the three-month follow-up, the authors identified significantly higher mean grades of anterior DL injury in the high-grade unstable group compared to the stable and low-grade stable groups (P = 0.037 and 0.004, respectively). However, they found no significant difference in MCS measurements between the groups. They concluded that valgus instability may persist after lateral malleolar fixation and that repair of the anterior DL is adequate in limiting postoperative lateral talar excursion [24].

Little et al. retrospectively analyzed 45 SER-IV equivalent ankle fracture patients with evidence of syndesmotic instability, DL, and posterior-inferior tibiofibular ligament (PITFL) injury on MRI (25). They compared TS screw fixation to combined DL and PITFL repair. Eighteen patients were treated with ORIF and TS screw fixation, while 27 underwent DL and PITLF repair. Measured outcomes included syndesmotic reduction based on MCS, tibiofibular clear space (TCS) measurements and range of motion. Follow-up was maintained for a minimum of one year. Two patients (7.4%) in the DL- and PITFL-fixation group and six (33.3%) in the TS-fixation group had syndesmotic malreduction (P = 0.002). At the final follow-up, the MCS in the anatomic fixation group was 2.5 mm. This measurement was significantly lower than that in the TS-fixation group, which was 3.5 mm (P = 0.005). However, they did not describe any significant mean change in TCS or MCS between the two cohorts. Although not clinically significant, they described a statistically significant difference in dorsiflexion in the TS cohort at final follow-up (20 vs 17 degrees, P = 0.02). Fourteen patients (78%) in the TS-fixation group and three patients (11%) in the anatomic-fixation group underwent implant removal. Notably, the authors wrote that there was a higher proportion of tobacco smokers in the TS repair group (five vs one: P = 0.02). This study demonstrated that PITFL and DL repair improves immediate postoperative outcomes while eliminating the need for reoperation for TS implant removal [25].

Studies Not Supporting Routine DL Repair

Stromsoe et al. performed a randomized blinded study to examine whether repair of a ruptured DL affected clinical outcomes [26]. They randomized 50 patients with lateral malleolar fractures (Weber Type B or C), suspected DL injury, and MCS widening on radiographic imaging into two groups of 25. Factors such as sex, age, fracture type, or presence of syndesmotic injury were similar between groups. One group underwent fibular ORIF alone (non-suture group), and the other, fibular ORIF with exploration and direct repair of the DL (suture group). The authors did not disclose the number of patients who underwent syndesmotic fixation. Intraoperative radiographs were used for confirmation of talar-mortise reduction. None of the patients in the non-suture group required exploration of the medial ligaments due to talar non-reduction. The final follow-up was performed at a mean of 17 months (range, 5-35 months). All radiographs in both groups showed normal fracture healing. No significant differences in hospital stay length, symptoms, and clinical findings were found between the two groups. Longer median duration of operation was found in the suture group (95 minutes) compared to the non-suture group (75 minutes). The authors concluded exploration and repair of a ruptured DL are unnecessary if anatomic reduction of the MCS is achieved [26].

Sun et al. analyzed 41 Weber Type B fracture patients with associated DL rupture (evidenced by MCS widening) and lateral/posterolateral talar dislocation [27]. After fixation of the ankle fracture, patients were assigned into three treatment groups depending on the trauma ward to which they were admitted. Patients admitted to Ward 1 underwent deep DL layer augmentation (n = 16); Ward 2, superficial DL repair (n = 12); and Ward 3, conservative management without DL repair (n = 13). The authors did not disclose the number of patients who underwent syndesmotic fixation. All patients maintained follow-up visits for a three-year minimum. Measured outcomes include plantar flexion and dorsiflexion of the affected ankle compared to the contralateral ankle, MCS, Philips and Schwartz, and AOFAS scores. No significant difference in range of motion or clinical outcomes was found between the three groups. They concluded that routine exposure, superficial repair, or deep augmentation of a ruptured DL is not indicated in Weber Type B fractures. Although the authors did not investigate this specifically, they speculated that DL augmentation may serve as a replacement for syndesmotic fixation in particular cases of Weber Type B and C fractures [27].

Discussion

Despite resulting in longer operation times, DL repair was shown to significantly decrease mean lengths of hospital stay [19]. Additionally, where some of the papers discussed in this study observed no additional benefit with DL repair, others noted significant improvement in clinical, functional, or radiologic outcomes [11]. Some indicated that DL repair may be a good alternative to TS screw fixation in ankle fracture patients with DL injury due to a lowered rate of malreduction and obviated the need for implant removal [22,23]. Results comparing ORIF with DL and PITFL fixation to ORIF with TS screw in SER-IV equivalent ankle fracture patients indicated that the former improved immediate postoperative syndesmotic reduction while obviating the need for implant removal [25]. Finally, DL repair has been shown to limit talar excursion and medial instability, benefiting patients with residual valgus instability after ORIF in isolated lateral malleolar fractures [24].

There is also evidence that the DL indirectly stabilizes the syndesmosis [10-12], thereby lending credibility to the idea that DL repair after ORIF and syndesmotic repair may benefit patients with concomitant DL-syndesmotic disruption [20]. Zhao et al. demonstrated that DL repair significantly reduces malreduction rates in Weber Type C fractures [21]. Furthermore, while they did not distinguish the Danis-Weber classification types of patients undergoing syndesmotic fixation, Weber Type C fractures are often associated with syndesmotic injury [28].

Lastly, some studies do not support routine DL repair [26,27]. While the limitations of each paper can be seen in Table 1, some notable weaknesses warrant discussion. Stromsoe et al. did not record whether the syndesmotic repair was performed in patients with concomitant syndesmotic-deltoid injury [26]. If the syndesmosis was indeed repaired, it may have masked the sequelae of an unrepaired DL. Sun et al. concluded that operative management of a ruptured DL was not necessary for Weber Type B fractures [27]. However, they stated that in some cases, medial augmentation could replace syndesmotic repair. Their conclusion was based on DL repair requiring greater attention to detail than syndesmotic fixation while achieving similar results.

**Table 1:** Literature reviewed on deltoid ligament (DL) injuries in ankle fractures. AOFAS = American Orthopaedic Foot and Ankle Society, LOE = level of evidence, MCS = medial clear space, ORIF = open reduction internal fixation, PER = pronation-external rotation, PITFL = posterior-inferior tibiofibular ligament, SER = supination-external rotation, TCS = tibiofibular clear space, VAS = visual analog scale

Study	Characteristics	Groups	Conclusion	Limitations
Stromsoe et al. [26]	- Weber type B and C fractures with radiographic evidence of DL injury (MCS widening)	- ORIF alone - ORIF and DL repair	- Ruptured DL can be left unexplored if anatomic MCS reduction is possible.	- None noted
Sun et al. [27]	- Weber type B fractures with DL rupture and lateral/posterior-lateral dislocation of the talus. - Measured outcomes: MCS, plantar and dorsiflexion, AOFAS scores, Philips and Schwartz scores	- ORIF alone - ORIF + superficial DL repair - ORIF + deep DL augmentation	- No indication for routine exposure and repair/augmentation of DL injuries in Weber type B fractures - DL augmentation can replacesyndesmotic fixation under certain circumstances	- Small sample size - No randomization - Comorbidities not recorded
Woo et al. [20]	- Closed SER and PER lateral malleolar fracture with DL injury - Measured outcomes: radiographic findings (MCS), AOFAS and VAS scores, foot function index (FFI)	- ORIF alone - ORIF + DL repair	- Medial instability may exist following ankle fracture fixation - Direct DL repair is adequate for restoring medial stability in high-grade unstable fractures with syndesmotic instability	- Retrospective design - Small sample size - Short follow-up period (17 mos.) - MRI and arthroscopy not performed routinely - Osteochondral lesion diagnosis and treatment not performed routinely - Consensus review performed by 2 readers with experience disparities
Zhao et al. [21]	- Closed Weber B and C fractures with evidence of DL injury (MCS>6mm) - Measured Outcomes: AOFAS and VAS scores as well as MCS measurements (preoperatively, postoperatively, and at final follow-up)	- ORIF alone - ORIF + DL repair	- Surgical DL repair can decrease postoperative MCS and malreduction rates, especially in Weber Type C fractures.	- Retrospective design - No random group assignment - MCS ≥ 6 mm on X-ray without stress or gravity-stress defined DL rupture - MCS ≥ 5 mm defined malreduction
Gu et al. [19]	- Ankle fractures with evidence of DL injury (MCS>5mm) -Measured Outcomes: AOFAS and VAS scores as well as postoperative MCS measurements.	- ORIF alone - ORIF + DL repair	DL repair can restore MCS, improve fracture healing and ankle function, as well as reduce chronic pain.	- Small sample size - Short follow-up period
Wu et al. [22]	- Weber type B and C ankle fractures with suspected DL injury (MCS >4mm and talus outward shifting. - Measured Outcomes: AOFAS, VAS, and SF-36 scores. MCS measurements	- ORIF + transsydesmotic screw fixation - ORIF + DL repair	-DL repair with suture anchor provided functional and radiologic outcomes comparable to those of screw fixation with a lower malreduction rate.	- Small sample size - Short follow-up period
Jones et al. [23]	- isolated SER-IV bimalleolar equivalent ankle fracture (Weber B fracture with MCS >5mm on stress test) - Measured outcomes: Lower Extremity Function Scale, Foot and Ankle Disability Index, Short Musculoskeletal Function Assessment, Foot and Ankle Outcome Score, AOFAS scores, VAS scores, and overall function of the lower extremity.	- ORIF + transsydesmotic screw fixation - ORIF + DL repair	DL repair can restore congruity to the ankle joint and has subjective, functional, and radiologic outcomes comparable to syndesmotic fixation while obviating the need to remove symptomatic implants in isolated SER-IV bimalleolar equivalent ankle fractures	- Retrospective design - Small sample size - Large number of surgeons performed surgical procedures - Several patients did not return outcome questionnaires
Lee et al. [24]	- Isolated malleolar fractures - Only patients with residual valgus instability following ORIF underwent DL repair - Measured Outcomes: Mean anterior deltoid ligament grade (MADLG), mean posterior deltoid ligament grade (MPDLG), and mean MCS for both injured and uninjured sides.	- Non-operative treatment - ORIF alone - ORIF + DL repair	-Valgus instability may exist even after repair of the fracture. -Anterior deltoid ligament repair is adequate in limiting postoperative talar excursion. -The anterior deltoid may contribute to medial stability more-so than what has previously been described.	- Small sample size - Short follow-up period - Only radiographic outcomes assessed - No comparison between high-grade unstable fracture patients who have undergone DL repair and those who have not
Little et al. [25]	- SER IV equivalent ankle fractures with ligamentous injury and syndesmotic instability. - Measured Outcomes: Postoperative CT showing syndesmotic reduction compared to the contralateral extremity, maintenance of reduction (based on MCS and TCS) on final postoperative radiograph	- ORIF + transsydesmotic screw fixation - ORIF + DL repair + PITFL repair	-Lateral malleolar fixation with DL and PITFL repair provided excellent radiographic outcomes in SER-IV equivalent ankle fractures without increasing postoperative complications, therefore eliminating the need for transsydesmotic screw fixation.	- Small sample size - No comparison made between the two groups in terms of functional outcomes - Some patients excluded to evaluate a homogenous group of patients

Because current literature is limited, definitive guidelines regarding operative indications for ankle fractures with DL injury remain undeveloped. However, our review indicates that there is a lower rate of malreduction associated with DL repair compared to TS screw fixation. This outcome may be because either (1) direct visualization and repair of the DL improves the likelihood of anatomic reduction compared to TS screw fixation under indirect visualization, or (2) many studies removed TS screws, possibly causing late displacement in the absence of DL repair. Moreover, our results show that DL repair after ORIF may benefit patients with concomitant DL-syndesmotic disruption, and also those with Weber Type C fractures. As a final point, operative management of DL injuries may be beneficial in patients with residual valgus instability following ORIF in isolated lateral malleolar fractures.

The strengths and limitations of this study are as follows. Given the limited literature regarding operative indications for DL rupture in acute ankle fracture, this review includes a small sample size. However, this also means that reviews such as this are an invaluable addition to understanding said operative conditions. Furthermore, incongruent datapoints between the reviewed papers mean that no significant statistical analysis could be performed. With this in mind, we could not evaluate the potential presence of bias within the papers reviewed here.

## Conclusions

Guidelines regarding the utility of DL repair in the setting of ankle fractures remains somewhat unclear. Our review found that in this setting, DL repair may benefit patients with concomitant DL-syndesmotic disruption, those with Weber Type C fractures, and patients with residual valgus instability following ORIF of isolated lateral malleolar fractures. Further studies may be conducted to expand upon the utility of DL repair.
